# The Role of Virtual Care on Access to Mental Health Counseling or Therapy to Reduce Health Inequities in the United States: Considerations on Age, Race, and Education

**DOI:** 10.1177/26924366251382425

**Published:** 2025-09-26

**Authors:** Coralia Vázquez-Otero, Jusung Lee

**Affiliations:** Department of Public Health, University of Texas at San Antonio, San Antonio, Texas, USA.

**Keywords:** mental health counseling, virtual care, major depressive disorder (MDD), health inequities, sociodemographics

## Abstract

**Aim:**

To examine an association between virtual care and mental health care use and whether there is any different pattern in the association across key demographic characteristics, specifically age, race/ethnicity, and educational attainment.

**Methods:**

A cross-sectional, observation study was conducted among the general population in the United States. This population-based study used data from the 2022 National Health Interview Survey. Main effect and interaction models using binary logistic regression were performed.

**Results:**

Of a total of 708 males and 1,238 females with symptoms of major depressive disorder (MDD), virtual care was associated with an increase in mental counseling or therapy use (males: adjusted odds ratio [aOR]: 2.75, 95% CI: 1.82–4.16, *p* ≤ 0.001; females: aOR: 3.38, 95% CI: 2.42–4.73, *p* ≤ 0.001). Females from a Hispanic background (aOR: 7.17, 95% CI: 2.97–17.33, *p* ≤ 0.001), aged 18–29 (aOR: 7.18, 95% CI: 3.51–14.70, *p* ≤ 0.001), and with less than high school graduation (aOR: 5.55, 95% CI: 1.97–15.62, *p* = 0.001) or college degrees (aOR: 5.52, 95% CI: 2.91–10.47, *p* ≤ 0.001) had notable increases in mental counseling or therapy use with virtual care.

**Conclusions:**

Virtual care is associated with a significant increase in accessing mental health care. Individuals who historically experience challenges related to in-person settings, including those with low educational attainment and Hispanics, may significantly benefit from virtual options.

## Introduction

In 2021, about 58 million adults in the United States had mental health conditions, and half of Americans were diagnosed with mental disorders at some point in their lifetime, suggesting that mental health is a major public health concern in the country.^1^ Moreover, recent data showed continued worsening in mental health with major depressive disorders (MDDs) rising from 6.6% in 2005 to 9.2% in 2020.^2,3^ Mental health conditions are associated with other public health issues such as substance use disorder, including drinking alcohol and smoking behaviors.^4,5^ For instance, people with mental conditions have higher smoking rates.^4^ Furthermore, previous work has pointed to the association between mental illness and substance use behaviors, including cigarette smoking and cessation across age groups and genders.^6^

To address these public health issues, the U.S. Healthy People 2030 has set goals that include timely prevention or interventions.^7^

Early, adequate interventions are critical, not only for improving mental health but also for additional benefits, such as enhancing social activities, including family relationships, daily routines, and work performances.^8^ Medications, such as antidepressants, are widely employed to prevent the onset of psychological symptoms or the advancement of existing conditions.^9^ Psychotherapy, another common form of mental health treatment, includes a range of measures, such as counseling and cognitive behavioral therapy.^10^ This approach, also known as talk therapy, has gained attention because clinical measures based on effective patient-provider communications and interactions facilitate evidence-based interventions that serve individuals’ care needs and increase clinical effectiveness.^11^ Despite these interventions, a national statistics report showed that only one in five U.S. adults receives mental health services in response to their health concerns.^12^

Considering the importance of mental health care, the existing evidence about gaps and variations in accessing mental health care across demographic characteristics is concerning.^13^ For example, less than half of Black individuals with moderate to severe symptoms of anxiety or depression (47%) reported receiving care, 0.73 times lower than their White counterparts (64%).^14^ Age-group differences have also been reported with younger adults receiving a higher rate of mental health care.^12^

Individuals who delay or miss necessary care complain about frequent barriers or challenges, such as time constraints, lack of mental health facilities, or difficulties making appointments with providers.^15,16^ With technological advancements, mental health services through virtual modalities have rapidly evolved. Compared with less than 1% of outpatient mental health care delivered in virtual settings in 2019, it became widely popular, rising to 35–40% between 2020 and 2021.^17^ Amid the health care disruptions during the COVID-19 pandemic, virtual care demonstrated a pivotal role in serving populations who may otherwise have delayed or foregone needed mental health services. Clinic-based studies also showed that virtual mental health care was not different in clinical outcomes and was associated with increased perceived quality of care compared with in-person care.^18,19^ Although most people report a preference for in-person care, they are open to virtual options.^20^ With unique features that either reduce or pose barriers, including availability and knowledge of technology and individuals’ attitudes, virtual care can have important implications for mental health care across populations.^20–22^

Despite prior studies documenting evolving virtual care across health care disciplines as alternatives to traditional face-to-face care, it is unclear how virtual options play a role in access to mental health care across varying population characteristics.^17–19^ Therefore, the current study aimed to 1) examine whether a significant association exists between an individual’s virtual care use status and mental health care use and 2) whether there is any different pattern in the association across key demographic characteristics, specifically age, race/ethnicity, and educational attainment. This study hypothesizes that virtual care use status is associated with increases in mental health care use, and there is heterogeneity in the association across population subgroups.

## Methods

### Data

This population-based, cross-sectional study used 2022 data from the National Health Interview Survey (NHIS), an annual household interview survey conducted by the U.S. Census Bureau.^23^ This population survey targets non-institutionalized adult individuals 18 years or older residing in 50 U.S. states and the District of Columbia. NHIS employs geographically clustered sampling techniques to select the sample and uses a face-to-face interview format to collect respondents’ self-reported health-related information. These data are widely used to monitor a broad range of national-level population health issues. Samples included in this study represent males and females who reported their symptoms of depression through the Patient Health Questionnaire (PHQ) 8 (Table 1) and reported whether they used mental health counseling or therapy. Of a total of 27,648 individuals, 1,948 participants who had PHQ 8 scores ≥ 10 (5% of males and 9.1% of females), considered as having symptoms of MDD, were included.^24^ Additional inclusion criteria selected participants who reported counseling or therapy experiences, resulting in 1,946 individuals for analysis (708 males and 1,238 females). As this study analyzed deidentified, publicly available secondary data, consent for the data was not necessary.

**Table 1.** Patient Health Questionnaire (PHQ)−8

How often during the past 2 weeks were you bothered by. . . .	Not at all	Several days	More than half the days	Nearly every day
1. Little interest or pleasure in doing things	0	1	2	3
2. Feeling down, depressed, or hopeless	0	1	2	3
3. Trouble falling or staying asleep, or	0	1	2	3
4. Feeling tired or having little energy	0	1	2	3
5. Poor appetite or overeating	0	1	2	3
6. Feeling bad about yourself, or that you are a failure, or have let yourself or your family down	0	1	2	3
7. Trouble concentrating on things, such as reading the newspaper or watching television	0	1	2	3
8. Moving or speaking so slowly that other people could have noticed. Or the opposite — Being so fidgety or restless that you have been moving around a lot more than usual	0	1	2	3

### Measures

#### Key outcome

The study outcome was a binary measure (Yes/No) of receiving mental health counseling or therapy using responses to the following question, “During the past 12 months, did you receive counseling or therapy from a mental health professional such as a psychiatrist, psychologist, psychiatric nurse, or clinical social worker?”

#### Covariates

The primary independent variable was an indicator of virtual care of any kind that participants reported through the following question, “During the past 12 months, have you had an appointment with a doctor, nurse, or other health professional by video or by phone?”

Key demographic characteristics of interest were age, race/ethnicity, and education, each categorized as 18–29, 30–39, and 40–49; non-Hispanic White, non-Hispanic Black, non-Hispanic others, and Hispanics; and less than high school graduation, high school graduation, some college education, and a bachelor’s degree or above, respectively. Additional covariates were included to account for potential confounding effects in the association, such as health insurance coverage, marital status, smoking status, general health, diabetes, and disability.^22,25,26^ Marital status was a dichotomous variable to indicate whether participants were married or not. Smoking status had three categories, such as never, formerly, and currently smoking. Health-related variables were dichotomized for good health or not good health and whether respondents had health conditions, including diabetes and disability.

### Statistical analysis

We calculated the percentage of mental health counseling or therapy use across participants’ demographic characteristics and then broke down this percentage by virtual care use. Binary logistic regression analysis was performed to examine an association between mental health counseling or therapy and virtual care use status without and with accounting for study covariates, such as age, race/ethnicity, education, marital status, health insurance, smoking, general health, diabetes, and disability. Since the interaction effect of virtual care on mental health care use is of primary interest, we calculated the percentage of people receiving counseling or therapy across age, race/ethnicity, and education by virtual care to assess whether there are differences in mental health care use associated with virtual care. Next, an interaction model using logistic regression was performed by interacting age, race/ethnicity, and education with virtual care (*virtual care use* × *age*, *virtual care use* × *education, and virtual care use* × *race/ethnicity)* to evaluate any difference in mental health care use based on the individuals’ virtual care use.

In addition, sensitivity analysis was performed with the sample restricted to those with symptoms of severe MDDs (PHQ-8 scores ≥ 20) to examine if the associations are consistent with those of primary analyses. Complex survey weights and design variables were adjusted in all analyses. The statistical significance level was *p* < 0.05 and STATA version SE 18.0 was used. This study was reviewed by the University of OO Institutional Review Board and additional ethical approval was not necessary because the data are part of publicly available secondary datasets.

## Results

Of a total of 708 males with symptoms of MDD, about 41.1% received mental health counseling or therapy (Table 2). This rate decreased with age increase, going from 49.8% (ages 18–29) to 31.7% (ages 65+). Those with some college education or above had a higher rate of receiving mental health care compared with those with less than high school graduation (39.1∼47.9% vs. 28.8%). Among females with symptoms of MDD, about 42.4% reported that they received mental health care with adults aged 18–39 and those with some college education or above revealed a disproportionately higher rate. In both males (55.9% vs. 28.3%) and females (55% vs. 26.3%), mental health counseling or therapy was more prevalent for those who use virtual care compared with those who do not.

**Table 2.** Mental Health Counseling or Therapy Use (%) by Sample Characteristics in Males and Females with Symptoms of Major Depressive Disorders

	Males			Females		
	*n* (wt %)	Counseling or therapy *n* (wt %)	*p*-Value	*n* (wt %)	Counseling or therapy *n* (wt %)	*p*-Value
Overall	708 (38.1)	287 (41.1)		1,238 (61.9)	520 (42.4)	
Virtual care			<0.001			<0.001
No	380 (53.6)	100 (28.3)		558 (43.9)	148 (26.3)	
Yes	328 (46.4)	183 (55.9)		679 (56.1)	372 (55.0)	
Age			0.014			<0.001
18–29	122 (24.8)	66 (49.8)		212 (27.1)	120 (55.6)	
30–39	116 (16.0)	59 (48.4)		188 (15.7)	111 (54.3)	
40–49	99 (14.9)	41 (44.9)		180 (14.8)	77 (38.8)	
50–64	202 (26.3)	71 (32.9)		349 (25.3)	150 (40.0)	
65+	169 (18.0)	50 (31.7)		309 (17.1)	62 (17.2)	
Race/ethnicity			0.137			0.284
White	476 (63.5)	197 (43.7)		855 (65.9)	372 (44.6)	
Black	80 (12.9)	27 (29.4)		141 (12.2)	55 (40.2)	
Hispanic	103 (17.0)	41 (34.8)		171 (15.7)	44 (36.7)	
Others	49 (6.6)	22 (45.3)		71 (6.2)	27 (37.9)	
Education			0.027			<0.001
<High school graduation	104 (17.1)	26 (28.8)		155 (14.7)	43 (30.8)	
High school graduation	216 (34.2)	81 (39.1)		342 (28.4)	126 (37.2)	
Some college	217 (31.4)	105 (47.9)		433 (35.3)	189 (45.5)	
≥College degree	166 (17.3)	73 (45.5)		303 (21.6)	160 (52.4)	
Marital status			0.018			0.026
Not married	67 (12.2)	20 (26.8)		78 (7.1)	23 (28.4)	
Married	636 (87.8)	264 (43.0)		1,157 (92.9)	495 (43.4)	
Others			0.942			0.011
Health insurance	425 (55.4)	175 (42.1)		746 (54.3)	333 (46.7)	
No	205 (33.8)	81 (40.6)		348 (33.3)	126 (35.0)	
Yes	53 (10.8)	22 (40.3)		113 (12.5)	48 (42.4)	
Smoking status			0.651			0.469
Never	303 (45.0)	132 (43.9)		635 (57.7)	259 (42.7)	
Former	219 (31.5)	88 (39.8)		335 (23.1)	138 (39.2)	
Current	174 (23.5)	65 (39.4)		261 (19.2)	119 (44.9)	
General health			0.244			<0.001
Not good	371 (49.2)	138 (38.4)		619 (46.5)	216 (36.3)	
Good	337 (50.8)	149 (43.8)		619 (53.5)	304 (47.7)	
Diabetes			0.534			0.096
No	578 (82.0)	242 (41.8)		1,029 (85.2)	449 (43.5)	
Yes	129 (18.0)	45 (38.2)		207 (14.8)	70 (36.1)	
Disability			0.441			0.192
No	432 (61.3)	172 (39.8)		743 (63.2)	337 (44.0)	
Yes	276 (38.7)	115 (43.3)		495 (36.8)	183 (39.6)	

Simple logistic regression confirmed that those who use virtual care had greater odds of receiving counseling or therapy compared with those who do not use virtual care in males (odds ratio [OR]: 3.21, 95% CI: 2.19–4.72, *p* ≤ 0.001) and females (OR: 3.42, 95% CI: 2.53–4.62, *p* ≤ 0.001) (Table 3). The adjusted regression analysis showed consistent results.

**Table 3.** Association Between Population Characteristics and Mental Health Counseling or Therapy Utilization in Males and Females with Symptoms of Major Depressive Disorders

	Males		Females	
	OR [95% CI]	aOR [95% CI]	OR [95% CI]	aOR [95% CI]
Virtual care status				
No	Ref.	Ref.	Ref.	Ref.
Yes	3.21 [2.19–4.72]^*^	2.75 [1.82–4.16]^*^	3.42 [2.53–4.62]^*^	3.38 [2.42–4.73]^*^
Key characteristics				
Age				
18–29		3.02 [1.48–6.16]^**^		7.15 [4.15–12.34]^*^
30–39		2.62 [1.38–4.96]^**^		7.27 [4.03–13.11]^*^
40–49		1.89 [0.95–3.75]		3.10 [1.81–5.32]^*^
50–64		1.15 [0.66–2.00]		3.66 [2.27–5.90]^*^
65+		Ref.		Ref.
Race/ethnicity				
White		Ref.		Ref.
Black		0.53 [0.30–0.95]^***^		0.85 [0.50–1.44]
Hispanic		0.71 [0.41–1.22]		0.78 [0.51–1.20]
Others		0.87 [0.34–2.20]		0.72 [0.38–1.38]
Education				
<High school graduation		Ref.		Ref.
High school graduation		1.25 [0.65–2.43]		0.91 [0.53–1.57]
Some college		1.48 [0.77–2.82]		1.26 [0.75–2.12]
≥College degree		1.20 [0.62–2.35]		1.62 [0.94–2.79]
Control factors				
Marital status				
Not married		Ref.		Ref.
Married		1.04 [0.67–1.61]		0.58 [0.41–0.83]^**^
Others		0.86 [0.40–1.85]		0.61 [0.37–1.01]
Health insurance				
No		Ref.		Ref.
Yes		1.85 [0.95–3.61]		1.45 [0.70–2.98]
Smoking status				
Never		Ref.		Ref.
Former		0.79 [0.48–1.30]		1.23 [0.86–1.75]
Current		0.92 [0.55–1.53]		1.61 [1.05–2.48]^***^
General health				
Not good		Ref.		Ref.
Good		1.16 [0.71–1.90]		1.24 [0.86–1.75]
Diabetes				
No		Ref.		Ref.
Yes		1.25 [0.73–2.14]		1.33 [0.84–2.09]
Disability				
No		Ref.		Ref.
Yes		1.48 [0.93–2.35]		1.14 [0.79–1.64]

aOR represents adjusted odds ratio, accounting for population characteristics.

^*^ *p* < 0.001.

^**^ *p* < 0.05.

^***^ *p* < 0.1.

Variations were observed in the rate of mental health counseling or therapy by virtual care use status across age, race/ethnicity, and educational attainment (Table 4). Among male participants, individuals in the age groups of 50–64 (46.6%) and 65 or above (37.3%) showed receiving mental health care less frequently with virtual care than younger people (64.6 − 70.4%). A lower rate of mental health care use was also shown among those with less than a high school graduation. Among females, people aged 18–24 and 30–39 revealed a higher rate of mental health care use associated with virtual care than those of other age groups (65.5 − 75.2% vs. 24.5–50.1%). A notable difference was found in receiving mental health care for non-Hispanic black people (45.4%%) compared with non-Hispanic White counterparts (56.8%).

**Table 4.** Mental Health Counseling or Therapy Utilization by Population Characteristics by Virtual Care Use Status Among Individuals with Symptoms of Major Depressive Disorders

	Mental health counseling or therapy utilization (%)	
	Males	Females
Overall	46.4%	56.1%
Key characteristics		
Age		
18–29	65.4%	75.2%
30–39	70.4%	65.5%
40–49	64.6%	**44.7%**
50–64	46.6%	50.1%
65+	**37.3%**	24.5%
Race/ethnicity		
White	60.0%	56.8%
Black	**44.4%**	**45.4%**
Hispanic	**50.0%**	55.3%
Others	**45.6%**	48.2%
Education		
<High school graduation	**40.8%**	49.6%
High school graduation	55.6%	45.0%
Some college	60.8%	56.5%
≥College degree	55.8%	66.3%
Control factors		
Marital status		
Not married	59.1%	59.5%
Married	50.7%	45.0%
Others	61.0%	61.8%
Health insurance		
No	59.8%	**49.6%**
Yes	55.4%	55.3%
Smoking status		
Never	59.9	54.5
Former	51.1	51.3
Current	56.4	61.4
General health		
Not good	45.9%	43.6%
Good	68.2%	64.7%
Diabetes		
No	55.9%	56.5%
Yes	56.1%	46.7%
Disability		
No	56.5%	58.0%
Yes	55.3%	50.3%

Bolded values represent that mental health counseling or therapy use within each category is statistically insignificant at a significance level of 0.05.

Interaction models in Table 5 showed that among White males and females, virtual care use status was significantly associated with an increase in counseling or therapy use (males: adjusted odds ratio [aOR]: 2.75, 95% CI: 1.82–4.16, *p* ≤ 0.001; females: aOR: 3.38, 95% CI: 2.42–4.73, *p* ≤ 0.001). Markedly, Hispanic female participants experienced 7.17 times greater odds of counseling or therapy use for those who use virtual care compared with those who do not (95% CI: 2.97–17.33; *p* ≤ 0.001). In terms of age, younger females aged 18–29 showed a substantial association (aOR: 7.18, 95% CI: 3.51–14.70, p ≤ 0.001). Females with less than high school graduation (aOR: 5.55, 95% CI: 1.97–15.62, *p* = 0.001) or a college degree (aOR: 5.52, 95% CI: 2.91–10.47, *p* ≤ 0.001) had notable increases in mental health care use with virtual care. Among males, people aged 30–39 (aOR: 4.95, 95% CI: 1.89–12.96, *p* = 0.001) and 40–49 (aOR: 4.64, 95% CI: 1.58–13.59, *p* = 0.005) had increased mental health care associated with virtual care. While results from sensitivity analysis with individuals with symptoms of severe MDD were largely consistent, females aged 18–29, of Hispanic ethnicity, and having a college degree showed even a larger increase in mental health care use associated with virtual care use (Tables 6 and 7).

**Table 5.** Interaction Effect of Virtual Care Use Status on the Association Between Population Characteristics and Mental Health Counseling or Therapy Receipt Among Males with Symptoms of Major Depressive Disorders—*Interaction Model*

	Males: aOR [95% CI]			Females: aOR [95% CI]		
	Model 1 *(Virtual care use × Age)*	Model 2 *(Virtual care use × Race/Ethnicity)*	Model 3 *(Virtual care use × Education*)	Model 1 *(Virtual care use × Age)*	Model 2 *(Virtual care use × Race/Ethnicity)*	Model 3 *(Virtual care use × Education*)
Key characteristics						
Virtual care use × Age						
18–29	2.07 [0.83–5.16]			7.18 [3.51–14.70]^*^		
30–39	4.95 [1.89–12.96]^**^			3.32 [1.59–6.93]^**^		
40–49	4.64 [1.58–13.59]^**^			1.92 [0.91–4.05]		
50–64	2.85 [1.37–5.91]^**^			2.50 [1.46–4.28]^**^		
65+	1.51 [0.66–3.47]			2.28 [1.04–4.97]^***^		
Virtual care use × Race/Ethnicity						
White		3.25 [2.00–5.28]^*^			3.32 [2.16–5.09]^*^	
Black		3.13 [0.91–10.78]			1.54 [0.65–3.67]	
Hispanic		2.30 [0.87–6.08]			7.17 [2.97–17.33]^*^	
Others		0.80 [0.18–3.62]			3.56 [0.81–15.68]	
Virtual care use × Education						
<High school graduation			1.59 [0.51–4.97]			5.55 [1.97–15.62]^**^
High school graduation			2.80 [1.34–5.85]^**^			2.14 [1.23–3.73]^**^
Some college			3.24 [1.67–6.26]^**^			3.06 [1.71–5.47]^*^
≥College degree			3.11 [1.48–6.55]^**^			5.52 [2.91–10.47]^*^
Age						
18–29	Ref.	Ref.	Ref.	Ref.	Ref.	Ref.
30–39	2.43 [0.98–6.05]	2.95 [1.45–6.00]^**^	3.11 [1.52–6.35]^**^	3.68 [1.53–8.88]^**^	7.08 [4.10–12.23]^*^	7.17 [4.15–12.39]^*^
40–49	1.42 [0.59–3.42]	2.66 [1.40–5.07]^**^	2.64 [1.39–5.01]^**^	5.75 [2.34–14.13]^*^	7.60 [4.19–13.80]^*^	7.35 [4.06–13.31]^*^
50–64	1.00 [0.33–3.03]	1.87 [0.93–3.75]	1.90 [0.96–3.77]	3.63 [1.47–8.98]^**^	3.18 [1.85–5.47]^*^	3.13 [1.81–5.40]^*^
65+	0.80 [0.38–1.71]	1.11 [0.64–1.95]	1.18 [0.67–2.08]	3.45 [1.56–7.62]^**^	3.68 [2.27–5.97]^*^	3.74 [2.30–6.09]^*^
Race/ethnicity						
White	Ref.	Ref.	Ref.	Ref.	Ref.	Ref.
Black	0.53 [0.30–0.96]^***^	0.55 [0.25–1.18]	0.52 [0.29–0.93]^***^	0.93 [0.54–1.59]	1.23 [0.58–2.61]	0.83 [0.49–1.42]
Hispanic	0.72 [0.41–1.26]	0.85 [0.43–1.67]	0.72 [0.42–1.25]	0.80 [0.52–1.24]	0.49 [0.22–1.10]	0.76 [0.49–1.18]
Others	0.86 [0.34–2.19]	1.57 [0.48–5.16]	0.88 [0.35–2.23]	0.72 [0.37–1.40]	0.70 [0.18–2.70]	0.75 [0.39–1.42]
Control factors						
Marital status						
Not married	Ref.	Ref.	Ref.	Ref.	Ref.	Ref.
Married	1.03 [0.66–1.61]	1.07 [0.69–1.65]	1.05 [0.68–1.63]	0.60 [0.42–0.85]^**^	0.57 [0.40–0.81]^**^	0.57 [0.40–0.81]^**^
Others	0.88 [0.40–1.93]	0.89 [0.42–1.89]	0.85 [0.39–1.83]	0.61 [0.37–1.02]	0.59 [0.36–0.98]^***^	0.63 [0.38–1.03]
Insurance						
No	Ref.	Ref.	Ref.	Ref.	Ref.	Ref.
Yes	1.91 [0.96–3.79]	1.85 [0.95–3.60]	1.88 [0.96–3.37]	1.47 [0.71–3.03]	1.37 [0.65–2.86]	1.51 [0.73–3.12]
Smoking status						
Never	Ref.	Ref.	Ref.	Ref.	Ref.	Ref.
Former	0.76 [0.46–1.25]	0.78 [0.69–1.65]	0.77 [0.47–1.28]	1.27 [0.90–1.80]	1.21 [0.85–1.73]	1.22 [0.85–1.74]
Current	0.95 [0.57–1.60]	0.89 [0.42–1.89]	0.92 [0.55–1.53]	1.60 [1.05–2.43]^***^	1.65 [1.07–2.55]^***^	1.60 [1.04–2.46]^***^
General health						
Not good	Ref.	Ref.	Ref.	Ref.	Ref.	Ref.
Good	1.17 [0.71–1.91]	1.16 [0.71–1.89]	1.18 [0.71–1.94]	1.22 [0.85–1.76]	1.26 [0.88–1.80]	1.26 [0.88–1.79]
Diabetes						
No	Ref.	Ref.	Ref.	Ref.	Ref.	Ref.
Yes	1.30 [0.76–2.21]	1.25 [0.72–2.17]	1.23 [0.71–2.10]	1.32 [0.85–2.05]	1.32 [0.84–2.09]	1.34 [0.85–2.11]
Disability						
No	Ref.	Ref.	Ref.	Ref.	Ref.	Ref.
Yes	1.47 [0.92–2.34]	1.51 [0.95–2.42]	1.50 [0.94–2.37]	1.14 [0.79–1.64]	1.18 [0.82–1.70]	1.16 [0.81–1.65]

aOR denotes adjusted odds ratio, accounting for population characteristics, and aOR of the interaction effect is the odds of receiving mental health counseling or therapy among those who reported virtual care use compared with odds of receiving mental health counseling or therapy among those with reported no virtual care use within each category of the population characteristic.

^*^ *p* < 0.001.

^**^ *p* < 0.05.

^***^ *p* < 0.1.

**Table 6.** Virtual Care Use by Population Characteristics in Males and Females with Severe Major Depressive Disorders (PHQ-8 Scores ≥20)

	Males (*n* = 708)		Females (*n* = 1,238)	
	%	*p*-Value	%	*p*-Value
Overall	328 (46.4)		679 (56.1)	
Age		0.794		0.063
18–29	53 (42.4)		121 (57.8)	
30–39	53 (46.4)		105 (56.7)	
40–49	51 (51.4)		110 (63.5)	
50–64	92 (46.3)		196 (56.3)	
65+	79 (57.7)		147 (46.3)	
Race/ethnicity		0.170		0.010
White	229 (49.1)		479 (59.5)	
Black	31 (34.9)		66 (42.1)	
Hispanic	46 (47.2)		92 (41.7)	
Others	22 (40.3)		42 (59.5)	
Education		<0.001		0.006
<High school graduation	30 (29.9)		68 (45.2)	
High school graduation	82 (38.7)		171 (42.7)	
Some college	114 (55.7)		246 (59.3)	
≥College degree	99 (60.5)		192 (63.4)	
Marital status		0.065		0.902
Not married	185 (42.7)		407 (56.7)	
Married	106 (53.8)		197 (57.6)	
Others	21 (39.4)		62 (55.0)	
Health insurance		<0.001		<0.001
No	13 (17.1)		18 (25.9)	
Yes	313 (50.5)		659 (58.4)	
Smoking status		0.097		0.442
Never	132 (44.6)		359 (57.7)	
Former	119 (54.0)		185 (54.8)	
Current	73 (41.6)		130 (52.4)	
General health		0.013		0.761
Not good	189 (52.0)		332 (55.6)	
Good	139 (40.9)		347 (56.6)	
Diabetes		0.593		0.281
No	264 (45.9)		559 (55.4)	
Yes	64 (48.8)		119 (60.3)	
Disability		0.021		0.191
No	184 (42.2)		400 (54.5)	
Yes	144 (52.9)		279 (58.9)	

**Table 7.** Association Between Population Characteristics and Receipts of Mental Health Counseling or Therapy in Males and Females with Symptoms of Severe Major Depressive Disorders (PHQ-8 Scores ≥20)

	Males	Females
	aOR [95% CI]	aOR [95% CI]
Virtual care		
No	Ref.	Ref.
Yes	2.70 [1.32–5.51]^*^	4.93 [2.78–8.75]^**^
Age		
18–29	3.05 [0.95–9.81]	10.14 [3.77–27.25]^**^
30–39	2.47 [0.88–6.99]	10.45 [3.66–29.83]^**^
40–64	1.32 [0.57–3.04]	5.88 [2.63–13.16]^**^
65+	Ref.	Ref.
Race/ethnicity		
White	Ref.	Ref.
Black	0.31 [0.11–0.91]^***^	1.00 [0.39–2.54]
Hispanic	0.45 [0.18–1.14]	1.14 [0.59–2.24]
Others	0.64 [0.17–2.35]	1.00 [0.26–3.90]
Education		
<High school graduation	Ref.	Ref.
High school graduation	2.21 [0.74–6.62]	0.98 [0.42–2.27]
Some college	1.81 [0.62–5.27]	1.60 [0.67–3.82]
≥College degree	1.72 [0.50–5.93]	2.46 [1.02–5.95]^***^
Control factors		
Marital status		
Not married	Ref.	Ref.
Married	0.91 [0.46–1.81]	0.38 [0.21–0.69]^***^
Others	0.34 [0.11–1.10]	0.36 [0.15–0.87]^***^
Smoking status		
Never	Ref.	Ref.
Former	1.55 [0.73–3.30]	1.12 [0.63–2.00]
Current	0.78 [0.35–1.76]	1.41 [0.73–2.75]
Health insurance		
No	Ref.	Ref.
Yes	0.92 [0.36–2.36]	3.10 [0.87–11.12]
General health		
Not good	Ref.	Ref.
Good	0.92 [0.36–2.36]	1.95 [1.11–3.43]
Diabetes		
No	Ref.	Ref.
Yes	1.63 [0.69–3.88]	1.36 [0.65–2.84]
Disability		
No	Ref.	Ref.
Yes	1.18 [0.59–2.36]	1.77 [1.03–3.07]

aOR represents adjusted odds ratio, accounting for population characteristics.

^*^ *p* < 0.05.

^**^ *p* < 0.001.

^***^ *p* < 0.1.

## Discussion

This study examined the relationship between virtual care and receiving mental health counseling or therapy, focusing on key demographic characteristics, such as age, race/ethnicity, and education. Between 41% and 43% of the study participants received mental health care and approximately half reported virtual care use. Virtual care use status was associated with the increased use of mental health counseling or therapy among both males and females, suggesting that virtual care may play an important role in improving access to mental health services and treatments.^19,27^ However, there were heterogeneous patterns in this association across assessed key population characteristics.

Specifically, males aged between 30 and 59 years old or with at least high school graduation had more frequent mental health care use associated with virtual care use status. Moreover, non-Hispanic White male participants showed increased mental health care use among those who use virtual care compared with those who do not, while non-White minorities did not show a significant association. These findings align with previous work and may suggest that since virtual care requires certain tools, including technologies and spaces, these subsets of the population may have better access to these resources, allowing them to address mental health care needs through virtual options.^28,29^ The findings may also reflect what has previously been reported in terms of knowledge or skills necessary for virtual care affirming that White, younger, highly educated individuals experience fewer issues than their racial minority counterparts, including competency in digital environments.^28,30,31^ Furthermore, perceptions about the wide-ranging advantages of virtual modalities, including less concerns about time and geographical limitations, are possibly contributing to this association.

Conversely, males with less than high school graduation, those aged 65 years or older, and racial and ethnic minorities did not show increases in receiving mental health care with virtual care. Previous research has shown that people with low educational attainment and older adults often complain about technology barriers and low levels of digital capability, undermining their ability to use technology-assisted care and the satisfaction associated with it.^21,32^ With such lacking skills and up-to-date technology support, it is possible that these individuals experience limited benefits out of virtual modalities or even disruptions of care, leading them to seek in-person visits or forgo care if in-person care is not accessible. While the evidence suggests that virtual care can positively influence health care access, the population variations raise substantial concerns about the possibility that certain groups of people continue to encounter barriers that prevent them from utilizing timely care assisted by virtual modalities as needed or wanted to fill health care gaps they experience.^18,19^

Overall, females who use virtual care showed greater use of mental health counseling or therapy. Specifically, younger people aged between 18 and 39 had a noted increase in mental health care use with virtual care. According to the national study, young females are the most vulnerable to psychological disorders.^33^ Their disproportionate exposure to many stressors related to household work and childcare responsibilities is among the probable factors causing poor mental health.^34^ Females in this age range are also characterized as individuals of reproductive age, suggesting that they face pregnancy-related stressors over their perinatal periods. Notably, depression is one of the most common maternal morbidities during prenatal and postpartum.^35^ However, despite the anticipated high mental care needs, burdens of family responsibilities and pregnancy circumstances may impose substantial restrictions on individuals’ time, travel, and physiological state in pursuing physical encounters with providers. These unique experiences may drive young females to look for mental health care remotely to substitute for in-person visits.^36,37^

In addition, females with a college degree revealed a marked increase in mental health care use given virtual care use, which is aligned with earlier findings that higher educational attainment is associated with higher levels of positive attitudes toward virtual options and is linked to fewer barriers.^38,39^ However, Hispanics and those having less than a high school graduation also showed improved access to mental health care when they were using virtual care. Earlier findings from a national study on women aged 18–44 suggest that those with lower educational attainment and from a Hispanic ethnic group tended to use virtual care more frequently than those with higher education levels and other racial/ethnic groups.^22^ Research has shown that these disadvantaged populations experience poor health care and challenges in clinical settings, which has been a major obstacle, triggering individuals to delay or forgo necessary care.^40,41^ Multi-faceted factors, such as language barriers, discrimination, and insurance denials concerning in-person visits, are potential determinants for these historically disadvantaged people to choose virtual options.^22,42–44^ These heterogeneous associations across populations are significant findings, advancing the knowledge base on mental health care in the rapidly changing health care landscape. Future studies are warranted to further the understanding of what specific circumstances lead to these observations.

### Policy implications

Virtual care may provide additional options for accessing quality mental health care. Health care professionals consider integrating in-person and virtual care as a promising opportunity to improve access to care across health care disciplines.^45^ Although the current analysis indicates that the general population can benefit from using virtual care, some may not do so due to barriers they face at both individual and community levels. Markedly, females having low educational attainment and Hispanic females had increases in mental health care use with virtual care, which further suggests that those who experience challenges related to in-person settings may benefit from virtual options. The dynamic associations found in this study require continued in-depth investigations into what influences people’s decisions regarding mental health care using information and communication technology. These findings provide important implications to inform health care and public health policy in efforts to improve Americans’ mental health. For instance, clinical and public health efforts to decrease substance use among individuals who suffer from mental conditions might benefit from incorporating virtual care as an option to address disparities in access to care.^5^ Indeed, the increased use of virtual services in the current health care delivery, some due to the COVID-19 pandemic, has shown the effectiveness of virtual treatments for substance use.^46,47^ In addition, virtual services are considered an alternative to in-person treatments for individuals with limited access to mental health and substance use health care, such as those living in rural communities.^5,48^

### Limitations

This study has many strengths, including analysis of population-representative data. However, this study has some limitations. First, while our findings showed increased mental health counseling or therapy for those who use virtual care, our key independent variable is not exclusive to mental health care. Future studies are warranted to improve or strengthen the current findings by analyzing more targeted measures. Second, this study relies on participants’ self-reports and inherently has limitations, such as social desirability and recall bias. Third, our study includes single cross-sectional data due to the availability of PHQ-8 questionnaires. An extended data period with continued collection of mental health screening would strengthen the current findings.

## Conclusions

Virtual care use is associated with an increase in accessing mental health counseling or therapy, revealing its potential to supplement traditional in-person care to tackle rising mental health problems. The current findings suggest the role of virtual care in improving access to mental health across population groups. It is critical to further the understanding of unique health care behaviors around mental health care and care modalities to better address health care access inequities.

## Data Availability

The data used in this analysis are publicly available from the National Health Interview Survey (NHIS)-https://www.cdc.gov/nchs/nhis/index.html.

## Abbreviations Used

ICT: Information and Communication Technology
MDD: Major depressive disorder
NHIS: National Health Interview Survey
OR: Odds ratio
PHQ: Patient Health Questionnaire
US: United States

## References

[1] National Institute of Mental Health. Mental Illness. Available from: https://www.nimh.nih.gov/health/topics/psychotherapies [Last accessed: January 10, 2025].

[2] Goodwin RD, Dierker LC, Wu M, et al. Trends in U.S. depression prevalence from 2015 to 2020: The widening treatment gap. Am J Prev Med 2022;63(5):726–733. 36272761 10.1016/j.amepre.2022.05.014 PMC9483000

[3] Weinberger AH, Gbedemah M, Martinez AM, et al. Trends in depression prevalence in the USA from 2005 to 2015: Widening disparities in vulnerable groups. Psychol Med 2018;48(8):1308–1315; doi: 10.1017/S0033291717002781 29021005

[4] Centers for Disease Control and Prevention. People with Mental Health Conditions. 2024. Available from: https://www.cdc.gov/tobacco/campaign/tips/groups/people-with-mental-health-conditions.html [Last accessed: February 2, 2025].

[5] Substance Abuse and Mental Health Services Administration. Key Substance use and Mental Health Indicators in the United States: Results from the 2019 National Survey on Drug Use and Health. Available from: https://www.samhsa.gov/data/sites/default/files/reports/rpt35325/NSDUHFFRPDFWHTMLFiles2020/2020NSDUHFFR1PDFW102121.pdf; 2021 [Last accessed: February 2, 2025].

[6] Lipari R, Horn SV. Smoking and Mental Illness among Adults in the United States. 2017. Available from: https://www.samhsa.gov/data/sites/default/files/report_2738/ShortReport-2738.html [Last accessed: February 2, 2025]. 28459516

[7] U.S. Department of Health and Human Services. Healthy People 2030. Available from: https://health.gov/healthypeople/objectives-and-data/browse-objectives/mental-health-and-mental-disorders [Last accessed: February 2, 2025].

[8] Martin C, Iqbal Z, Airey ND, et al. Improving Access to Psychological Therapies (IAPT) has potential but is not sufficient: How can it better meet the range of primary care mental health needs? Br J Clin Psychol 2022;61(1):157–174; doi: 10.1111/bjc.12314 34124792

[9] Unützer J, Tang L, Oishi S, et al.; IMPACT Investigators. Reducing suicidal ideation in depressed older primary care patients. J Am Geriatr Soc 2006;54(10):1550–1556; doi: 10.1111/j.1532-5415.2006.00882.x 17038073

[10] National Institute of Mental Health. Mental Illness. Available from: https://www.nimh.nih.gov/health/statistics/mental-illness#:∼:text=Mental%20illnesses%20are%20common%20in,of%20the%20U.S.%20adult%20population [Last accessed: February 10, 2025].

[11] Chowdhury P, Tari A, Hill O, et al. To improve the communication between a community mental health team and its service users, their families and carers. BMJ Open Qual 2020;9(4):e000914; doi: 10.1136/bmjoq-2020-000914 PMC7646348 33154096

[12] Terlizzi EP, Schiller JS. Mental health treatment among adults aged 18-44: United States, 2019-2021. NCHS Data Brief 2022(444):1–8. 36135999

[13] McGuire TG, Miranda J. New evidence regarding racial and ethnic disparities in mental health: Policy implications. Health Aff (Millwood) 2008;27(2):393–403; doi: 10.1377/hlthaff.27.2.393 18332495 PMC3928067

[14] Panchal N, Rae M, Saunders H, et al. How does use of Mental Health care vary by Demographics and Health Insurance Coverage?. 2023. Available from: https://www.kff.org/mental-health/issue-brief/how-does-use-of-mental-health-care-vary-by-demographics-and-health-insurance-coverage/#:∼:text=More%20than%20half%20(55%25),%2D64%3B%20and%2038%25%20for. [Last accessed: February 15, 2025]

[15] Lee J. Correlates of and disparities in cancellations or delays of prenatal visits during the Covid-19 pandemic: Emphasis on racial/ethnic minorities and persons with low socioeconomic status. J Racial Ethn Health Disparities 2024;11(3):1564–1577; doi: 10.1007/s40615-023-01632-3 37160575 PMC10169131

[16] Morales DA, Barksdale CL, Beckel-Mitchener AC. A call to action to address rural mental health disparities. J Clin Transl Sci 2020;4(5):463–467; doi: 10.1017/cts.2020.42 33244437 PMC7681156

[17] Lo J, Rae M, Amin K, et al. Telehealth has played an Outsized Role Meeting Mental Health needs during the COVID-19 Pandemic. 2022. Available from: https://www.kff.org/mental-health/issue-brief/telehealth-has-played-an-outsized-role-meeting-mental-health-needs-during-the-covid-19-pandemic/. [Last accessed: February 15, 2025].

[18] Bulkes NZ, Davis K, Kay B, et al. Comparing efficacy of telehealth to in-person mental health care in intensive-treatment-seeking adults. J Psychiatr Res 2022;145:347–352; doi: 10.1016/j.jpsychires.2021.11.003 34799124 PMC8595951

[19] Greenwood H, Krzyzaniak N, Peiris R, et al. Telehealth versus face-to-face psychotherapy for less common mental health conditions: Systematic review and meta-analysis of randomized controlled trials. JMIR Ment Health 2022;9(3):e31780; doi: 10.2196/31780 35275081 PMC8956990

[20] Mazurka R, Vallis EH, Chen L, et al. Preferences for virtual versus in-person mental and physical healthcare in Canada: A descriptive study from a cohort of youth and their parents enriched for severe mental illness. BMJ Paediatr Open 2024;8(1):e002197; doi: 10.1136/bmjpo-2023-002197 PMC10806455 38191204

[21] Betts LR, Hill R, Gardner SE. “There’s Not Enough Knowledge Out There”: Examining older adults’ perceptions of digital technology use and digital inclusion classes. J Appl Gerontol 2019;38(8):1147–1166; doi: 10.1177/0733464817737621 29165038

[22] Lee J, Manalew WS. Reasons for not pursuing virtual prenatal care in 2020 through 2021 and policy implications. Telemed J E Health 2023;29(10):1492–1503; doi: 10.1089/tmj.2022.0492 36787485 PMC10589501

[23] Centers for Disease Control and Prevention. National Health Interview Survey. Available from: https://www.cdc.gov/nchs/nhis/documentation/index.html

[24] Kroenke K, Strine TW, Spitzer RL, et al. The PHQ-8 as a measure of current depression in the general population. J Affect Disord 2009;114(1–3):163–173. 18752852 10.1016/j.jad.2008.06.026

[25] Cook BL, Wayne GF, Kafali EN, et al. Trends in smoking among adults with mental illness and association between mental health treatment and smoking cessation. JAMA 2014;311(2):172–182; doi: 10.1001/jama.2013.284985 24399556 PMC5555156

[26] Cree RA, Okoro CA, Zack MM, et al. Frequent mental distress among adults, by disability status, disability type, and selected characteristics - United States, 2018. MMWR Morb Mortal Wkly Rep 2020;69(36):1238–1243; doi: 10.15585/mmwr.mm6936a2 32914770 PMC7499832

[27] Ukert B, Lawley M, Kum HC. Geographic disparities in telemedicine mental health use by applying three way ANOVA on Medicaid claims population data. BMC Health Serv Res 2024;24(1):494; doi: 10.1186/s12913-024-10898-0 38649985 PMC11034036

[28] Mitchell UA, Chebli PG, Ruggiero L, et al. The digital divide in health-related technology use: The significance of race/ethnicity. Gerontologist 2019;59(1):6–14; doi: 10.1093/geront/gny138 30452660

[29] Ray R, Sewell AA, Gilbert KL, et al. Missed opportunity? leveraging mobile technology to reduce racial health disparities. J Health Polit Policy Law 2017;42(5):901–924; doi: 10.1215/03616878-3940477 28663182

[30] Hecker I, Briggs A. Overlooked and Under-Connected: Exploring Disparities in Digital Skill Levels Among Older Youth of Color in the US. Urban Institute; 2021. Available from: https://www.urban.org/research/publication/overlooked-and-under-connected-exploring-disparities-digital-skill-levels-among-older-youth-color-us [Last accessed: February 15, 2025].

[31] Miyawaki A, Tabuchi T, Ong MK, et al. Age and social disparities in the use of telemedicine during the COVID-19 pandemic in Japan: Cross-sectional Study. J Med Internet Res 2021;23(7):e27982; doi: 10.2196/27982 34259641 PMC8315162

[32] Adams RB, Nelson VR, Holtz BE. Barriers for telemedicine use among nonusers at the beginning of the pandemic. Telemed Rep 2021;2(1):211–216; doi: 10.1089/tmr.2021.0022 35720746 PMC9049802

[33] Collier Villaume S, Chen S, Adam EK. Age disparities in prevalence of anxiety and depression among US adults during the COVID-19 Pandemic. JAMA Netw Open 2023;6(11):e2345073; doi: 10.1001/jamanetworkopen.2023.45073 38032641 PMC10690464

[34] Aviv E, Waizman Y, Kim E, et al. Cognitive household labor: Gender disparities and consequences for maternal mental health and wellbeing. Arch Womens Ment Health 2025;28(1):5–14; doi: 10.1007/s00737-024-01490-w 38951218 PMC11761833

[35] Howard LM, Molyneaux E, Dennis CL, et al. Non-psychotic mental disorders in the perinatal period. Lancet 2014;384(9956):1775–1788; doi: 10.1016/S0140-6736(14)61276-9 25455248

[36] Bauman BL, Ko JY, Cox S, et al. Vital Signs: Postpartum depressive symptoms and provider discussions about perinatal depression - United States, 2018. MMWR Morb Mortal Wkly Rep 2020;69(19):575–581; doi: 10.15585/mmwr.mm6919a2 32407302 PMC7238954

[37] Brody DJ, Pratt LA, Hughes JP. Prevalence of depression among adults aged 20 and over: United States, 2013-2016. NCHS Data Brief 2018(303):1–8. 29638213

[38] Andersen JA, Rowland B, Gloster E, et al. Telehealth utilization during COVID-19 among people with diagnosed mental health conditions. Telemed J E Health 2022;28(5):743–746; doi: 10.1089/tmj.2021.0356 34515529 PMC9127827

[39] Tipre M, Scarinci IC, Pandya VN, et al. Attitudes toward telemedicine among urban and rural residents. J Telemed Telecare 2024;30(4):722–730; doi: 10.1177/1357633X221094215 35578537 PMC12947939

[40] Brach C, Fraser I. Reducing disparities through culturally competent health care: An analysis of the business case. Qual Manag Health Care 2002;10(4):15–28; doi: 10.1097/00019514-200210040-00005 12938253 PMC5094358

[41] Fryer K, Lewis G, Munoz C, et al. Identifying barriers and facilitators to prenatal care for Spanish-speaking women. N C Med J 2021;82(1):7–13; doi: 10.18043/ncm.82.1.7 33397748 PMC7927271

[42] Lee J, Howard KJ, Leong C, et al. Beyond being insured: Insurance coverage denial as a major barrier to accessing care during pregnancy and postpartum. Clin Nurs Res 2023;32(8):1092–1103; doi: 10.1177/10547738231177332 37264856 PMC10504817

[43] Sheppard VB, Williams KP, Wang J, et al. An examination of factors associated with healthcare discrimination in Latina Immigrants: The role of healthcare relationships and language. J Natl Med Assoc 2014;106(1):15–22; doi: 10.1016/S0027-9684(15)30066-3 26744111 PMC4838486

[44] Timmins CL. The impact of language barriers on the health care of Latinos in the United States: A review of the literature and guidelines for practice. J Midwifery Womens Health 2002;47(2):80–96; doi: 10.1016/s1526-9523(02)00218-0 12019990

[45] American Medical Association. Telehealth Survey Report. 2021. Available from: https://www.ama-assn.org/system/files/telehealth-survey-report.pdf [Last accessed: February 16, 2025].

[46] Fiacco L, Pearson BL, Jordan R. Telemedicine works for treating substance use disorder: The STAR clinic experience during COVID-19. J Subst Abuse Treat 2021;125:108312; doi: 10.1016/j.jsat.2021.108312 34016299 PMC8561322

[47] Lin LA, Casteel D, Shigekawa E, et al. Telemedicine-delivered treatment interventions for substance use disorders: A systematic review. J Subst Abuse Treat 2019;101:38–49; doi: 10.1016/j.jsat.2019.03.007 31006553

[48] Benavides-Vaello S, Strode A, Sheeran BC. Using technology in the delivery of mental health and substance abuse treatment in rural communities: A review. J Behav Health Serv Res 2013;40(1):111–120; doi: 10.1007/s11414-012-9299-6 23093443

